# Whole Brain 3D T1 Mapping in Multiple Sclerosis Using Standard Clinical Images Compared to MP2RAGE and MR Fingerprinting

**DOI:** 10.1002/nbm.70037

**Published:** 2025-04-15

**Authors:** Jeff Snyder, Gregg Blevins, Penelope Smyth, Alan H. Wilman

**Affiliations:** ^1^ Department of Radiology and Diagnostic Imaging University of Alberta Edmonton Alberta Canada; ^2^ Department of Biomedical Engineering University of Alberta Edmonton Alberta Canada; ^3^ Department of Medicine, Division of Neurology University of Alberta Edmonton Alberta Canada

**Keywords:** FLAIR, MP2RAGE, MR fingerprinting, qMRI, quantitative imaging, reconstruction, T1 mapping

## Abstract

Quantitative T1 and T2 mapping is a useful tool to assess properties of healthy and diseased tissues. However, clinical diagnostic imaging remains dominated by relaxation‐weighted imaging without direct collection of relaxation maps. Dedicated research sequences such as MR fingerprinting can save time and improve resolution over classical gold standard quantitative MRI (qMRI) methods, although they are not widely adopted in clinical studies. We investigate the use of clinical sequences in conjunction with prior knowledge provided by machine learning to elucidate T1 maps of brain in routine imaging studies without the need for specialized sequences. A classification learner was trained on T1w (magnetization prepared rapid gradient echo [MPRAGE]) and T2w (fluid‐attenuated inversion recovery [FLAIR]) data (2.6 million voxels) from multiple sclerosis (MS) patients at 3T, compared to gold standard inversion recovery fast spin echo T1 maps in five healthy subjects, and tested on eight MS patients. In the MS patient test, the results of the machine learner‐produced T1 maps were compared to MP2RAGE and MR fingerprinting T1 maps in seven tissue regions of the brain: cortical grey matter, white matter, cerebrospinal fluid, caudate, putamen and globus pallidus. Additionally, T1s in lesion‐segmented tissue was compared using the three different methods. The machine learner (ML) method had excellent agreement with MP2RAGE, with all average tissue deviations less than 3.2%, with T1 lesion variation of 0.1%–5.3% across the eight patients. The machine learning method provides a valuable and accurate estimation of T1 values in the human brain while using data from standard clinical sequences and allowing retrospective reconstruction from past studies without the need for new quantitative techniques.

## Graphical abstract

This work explores quantitative T1 map production in MS patients with machine learning using standard clinical 3D sequences (MPRAGE/FLAIR), with comparison to gold standard (IR), MP2RAGE and MRF. The method showed excellent agreement with MP2RAGE T1 values and is a viable substitute in the absence of dedicated T1 mapping sequences.

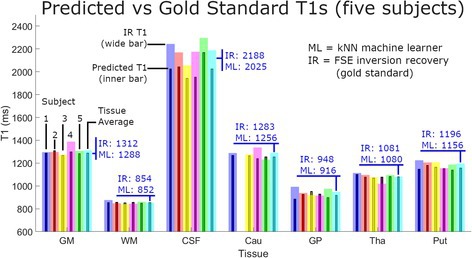

**Abbreviations**
B1^+^
: magnetic field (transmit)
Cau: caudate
CSF: cerebrospinal fluid
FLAIR: fluid‐attenuated inversion recovery
FSE: fast spin echo
GM: grey matter
GP: globus pallidus
IR: inversion recovery
KNN: k‐nearest neighbour
ML: machine learning or machine learner
MP2RAGE: dual‐echo magnetization prepared rapid gradient echo
MPRAGE: magnetization prepared rapid gradient echo
MRF: magnetic resonance fingerprinting
MS: multiple sclerosis
Put: putamen
qMRI: quantitative MRI
SPM: statistical parameter mapping
SSIM: structural similarity index
T1: longitudinal relaxation time
T1w: T1‐weighted
T2: transverse relaxation time
T2*: observed T2 (T2 + magnetic field inhomogeneities)
T2w: T2‐weighted
TA: acquisition time
TE: echo time
TI: inversion time
TR: repetition time
WM: white matter

## 1 Introduction

Quantitative MRI (qMRI) has emerged in recent years to provide standardized results of tissue properties in normal and diseased tissue [1, 2, 3, 4, 5]. While quantification of tissue parameters allows repeatable comparison, weighted images are still most prevalent in typical clinical exams, which provide different probes of tissue composition based on contrast, with T1‐ and T2‐weighting being the most common. Though MP2RAGE [6], MR fingerprinting (MRF [7, 8]) and other fast qMRI methods [9, 10] are rapidly gaining popularity, the main hurdles to acceptance of qMRI in many clinics are the increased time required for specialized qMRI sequences, the resistance to replacement of standard T1w and T2w sequences with novel multi‐purpose techniques and the need for (sometimes lengthy) offline reconstruction of qMRI maps [11, 12, 13]. Further, advanced sequence modeling and knowledge of machine learning or regression is required to produce accurate quantitative maps from base images, leading to specific expertise that may not be readily available in the clinic. In this study, we seek to remove the requirement of specialized qMRI sequences by constructing T1 maps in brain from common clinical sequences with using easily explainable machine learning methods.

The motivation for our study is the retrospective construction of T1 maps from standard clinical protocols that may not contain specific specialized T1 mapping sequences. This study focused on available data from inversion recovery (IR) 3D T1w volumetric imaging, also called magnetization prepared rapid gradient echo (MPRAGE [14]) and 3D fluid‐attenuated inversion recovery (FLAIR [15]). These two sequences are common in neurological 3D imaging protocols for T1‐ and T2‐weighted analysis, providing excellent contrast between white matter (WM) and grey matter (GM) in a 3D volume with clear lesion‐to‐tissue discrimination. For example, in multiple sclerosis (MS), hyperintense T2‐weighted lesions on FLAIR and hypointense T1‐weighted lesions on MPRAGE are common signatures. Utilizing clinical MS studies in brain and a second set of 11 MS patients with additional T1 maps from MP2RAGE [6] (dual‐echo MPRAGE with two inversion times), a machine learner (ML) was trained to produce T1 maps, with learner verification performed in five healthy subjects with gold standard inversion recovery fast spin echo (IR FSE) T1 maps. To evaluate efficacy in MS patients, the learner was subsequently tested on eight MS patients. Previous work using machine learning approaches have used synthetic data for training [16] and convolutional neural networks with multitasking sequences as references [17]. In contrast, this work explores an established classification learner using acquired data and validated against gold standard inversion recovery spin echo methods. While more complicated approaches from both sequence and processing standpoints exist, the methods used here are accessible to practically all researchers. This allows retroactive T1 map creation from existing clinical data using standard sequences and widely available machine learning programmes.

## 2 Methods

The study consisted of three stages: (1) training of the ML using MS patient data including MP2RAGE T1 maps, (2) validation of the ML in healthy subjects as compared to gold standard IR FSE T1 maps and (3) evaluation of the ML‐produced T1 maps in eight MS patients as compared to maps from MP2RAGE and MRF.

### 2.1 In Vivo Brain Experiments

All data were acquired using a Siemens Prisma (Erlangen, Germany) 3T scanner with an 80‐mT/m gradient set and a 64‐channel head and neck array for signal reception. Eleven MS patients (ages in years: 61M, 59M, 50F, 43F, 44M, 32F, 35F, 29F, 39F, 19F and 46F) and five healthy volunteers (ages 32F, 35F, 31M, 28M and 31F) were scanned after informed consent. For the ML model, the first three MS patients were used for training and the latter eight for evaluation. MS patients were chosen randomly from a larger patient cohort with initial screening of image quality only. All protocols included 3D MPRAGE and 3D FLAIR. MP2RAGE and MRF were additionally acquired in the MS patients and IR FSE in the healthy volunteers. Both MPRAGE and FLAIR were acquired sagittally using a 1‐mm^3^ isotropic resolution and 232 × 256 × 176 matrix size. Additional MPRAGE parameters consisted of TE = 2.2 ms, TI = 880 ms, an echo train length of 144 (4.5 ms echo spacing) TA = 3.6 min, with FLAIR using TE = 374 ms, TR = 5000 ms, TI = 1800 ms and a 247 echo train length (TA = 5.8 min). Parameters for the product 2D‐MRF sequence were: 2D‐steady‐state free precession (SSFP), variable flip angles as previously described [18], TE/TR = 2/12 ms, TI = 20.64 ms, 1‐mm^2^ in‐plane resolution (256 × 256) with 4.5‐mm slice thickness (14 total slices, TA = 4 min 50 s). MRF T1 map production was performed automatically using the built‐in scanner console software. The MP2RAGE parameters were 1‐mm^3^ isotropic resolution (232 × 256 × 160), TE/TR = 2.98/1500 ms, TI1/TI2 = 700/2500 ms, TA = 6 min 21 s. Single‐slice IR FSE experiments were conducted using seven different IR times (seven single‐slice images in total) with TE = 10 ms, TR = 7 s, 0.9‐mm^2^ in‐plane resolution, and 3‐mm slice thickness, with the slice located axially in iron‐rich deep GM (putamen, caudate, globus pallidus). Each IR image took 2.0 min, with inversion times of 50, 100, 200, 500, 1000, 2200 and 3000 ms (13.8 min total). All images were registered to MPRAGE using Statistical Parameter Mapping 12 (SPM12) [19, 20, 21]. Additionally, FLAIR images were bias‐field corrected using the N4 correction method in 3D Slicer [22, 23].

### 2.2 Training the ML for T1 Map Production

MPRAGE and FLAIR data were used as inputs and MP2RAGE T1 maps as outputs to a nearest‐neighbour (kNN) classification learner based in MATLAB (Natick, MA), with classes in the range T1 = 400 to 3980 ms in 10 ms increments. The kNN algorithm is one of the simplest machine learning methods and has been used extensively in disease detection [24, 25]. kNN is considered a lazy algorithm, in that the training phase consists merely of storing the training data. Therefore, a large subset of training data is required to properly represent all scenarios that are presented to the ML for prediction. As the use case for this learner is the production of T1 maps in disease with WM lesions, the training data consisted of a subset of the MS patient cohort (three full datasets). Data were restricted to regions in the brain with a large WM presence to enable proper lesion classification and therefore voxels inferior to the hippocampus in the axial direction were not included. In total, 2.6 million unique voxels were used for input data. In addition to the MPRAGE and FLAIR features, tissue probability maps were computed from MPRAGE data using SPM12 [19, 20, 21] for seven distinct regions (WM, GM, cerebrospinal fluid [CSF], caudate [Cau], globus pallidus [GP], thalamus [Tha] and putamen [Put]) to further delineate training voxels. These maps were then used as extra dimensions in the training data, resulting in nine inputs (the input vector) for mapping to a single T1 value from the MP2RAGE T1 map voxel. Essentially, for each T1 map value from MP2RAGE, there are nine inputs that help with the classification. While classification could be made using a single input (e.g. MPRAGE) by assigning T1 values to MPRAGE intensities, additional inputs aid in restricting the type of input voxel. Typically, in regression‐based fitting, two points are required along a simulated decay curve to calculate a T1 or T2 value from theory [1, 11], with additional echoes used to ensure accuracy (multiecho spin echo, in the case of T2 mapping). This is similar to the kNN approach—additional input points further determine the tissue composition that is being classified. Utilizing MPRAGE provides a direct correlation to T1 signal dependence, and the addition of FLAIR adds T1 and T2 variability, with tissue segmentation used to delineate between tissues with similar MPRAGE and FLAIR values. To ensure minimal intersubject variability of signal intensities, the MPRAGE and FLAIR images were normalized on a per volume and per subject basis, by dividing by the average WM value in each volume. Normalization provided consistent MPRAGE and FLAIR values for the training data (signal levels did not fluctuate between patients). Consequently, all predicted T1 maps required normalization of MPRAGE and FLAIR inputs to their own individual average WM signal intensities.

During the predictive or inference stage of the kNN learner (when T1 maps are produced), the input vector (consisting of the nine features listed above) is compared to the training data to make a classification (i.e. predict the T1 value). The inputs are compared to the closest k neighbours in the training data using a distance metric, and the classification is made based on the shortest distance. For example, in the simplified case of using only MPRAGE signal intensity as the input vector, the distance (intensity) between the input voxel, s_in_, is compared to the closest k neighbours of MPRAGE signal intensities in the training data, S_train_ and differences are recorded for each (s_in_—S_train,k_). The smallest difference found is used to classify S_in_ by assigning the T1 value associated with S_train,n_ from the training set. Expanding on this model, the distances for each element of the nine‐element input vector are compared to the training data with distances computed on element by element basis. After the classification is made, the learner progresses through each vector of the input to fully classify the data. To further optimize prediction, an iterative approach using a weighting matrix can be used to penalize distinct regions prone to misclassification (cost matrix). Typically, the cost matrix can help in difficult to classify regions that may result from similar input vectors with different output classes. To maintain the simple approach presented in this study, the distance metric used was the Euclidean distance (L2 norm as presented above for the simplified MPRAGE case) with the neighbourhood including one nearest neighbour (a ‘fine’ kNN model). Though not presented, including more than one neighbour tended to lose small features in the resultant T1 map. Further, a modified cost matrix was not employed (i.e. all misclassifications had identical penalties = 1).

### 2.3 Validating the ML T1 Maps in Healthy Volunteers

Subsequent to ML training with MS patient data, data from five healthy volunteers were used to validate the predicted T1 maps against gold standard maps produced from IR FSE. The single‐slice IR images were acquired with seven different inversion times, and regression‐based fitting was performed to extract the T1 value for each pixel based on an exponential recovery in the IR series. The axial slice data were acquired in an iron‐rich deep GM region to include many different tissue types. This T1 map was then compared to the predicted T1 map from the kNN ML algorithm, with data preparation of the input vector constructed in a similar fashion to the training data. Statistics included direct comparison of means for each tissue type, percent differences, structural similarity indices (SSIM) and Bland–Altman [26, 27] analysis.

### 2.4 Comparison of ML T1 Maps to MRF and MP2RAGE in MS Patients

The final stage consisted of ML prediction of T1 maps for eight MS patients. These maps were then compared with similar methods as used in healthy volunteers to MRF and MP2RAGE. Input data to the ML were prepared as before, and results were based on tissue type delineated by the tissue probability maps. While not used as a tissue segment in the training, tissue types were further divided to include a lesion group, based on semi‐automated segmentation of FLAIR images based on thresholding. Note that this is a further division of other tissue types with lesion T1 values already present in GM, WM and CSF based on the previous segmentation. The lesion group was used to highlight the use of the ML learner to investigate predicted T1 values in regions not found in healthy tissue.

## 3 Results

### 3.1 Validating the ML T1 Maps in Healthy Volunteers

**FIGURE 1:**
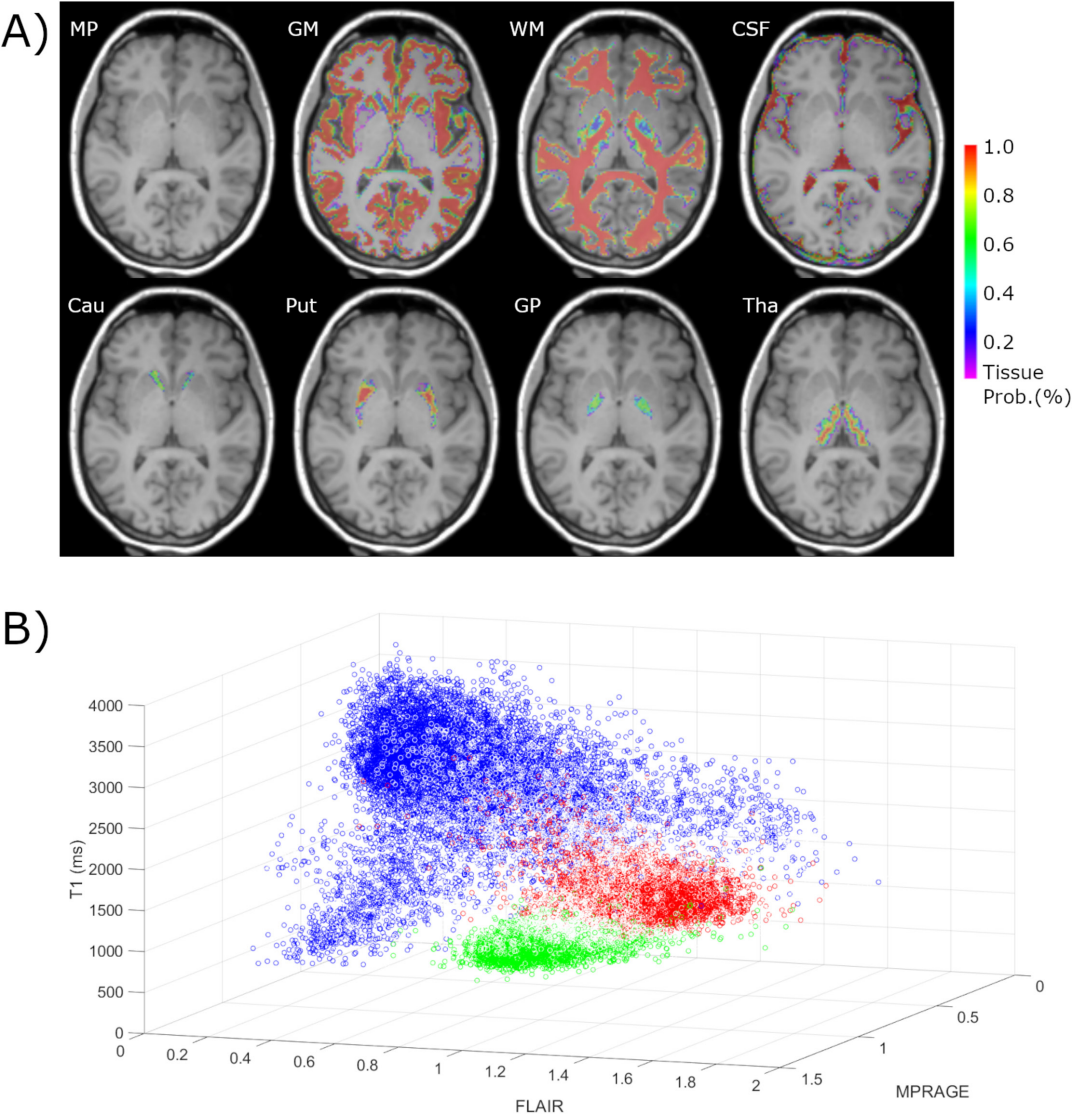
(A) Tissue probability maps overlaid on the reference MPRAGE image at the location of the IR FSE acquisition for healthy volunteer #1. (B) Data distribution for FLAIR and MPRAGE inputs (x‐ and y‐axes, respectively) with ML output (T1, z‐axis). Tissue probabilities > 0.95 are colour coded for GM (red), WM (green) and CSF (blue). CSF has the greatest spread in values, while GM and WM form regional groupings. Abbreviations used: Cau, caudate; GM, grey matter; GP, globus pallidus; CSF, cerebrospinal fluid; MP, MPRAGE; Put, putamen; Tha, thalamus; WM, white matter.

Figure 1A illustrates the slice location (MPRAGE) used for IR FSE for healthy volunteer #1 with the tissue probability maps overlaid. In Figure 1B, a scatter plot of data (x‐axis, MPRAGE; y‐axis, FLAIR) for the slice is shown to display the spread of the values occurring in the images. Tissue probabilities > 0.95 are colour coded for GM (red), WM (green) and CSF (blue). Each point indicates the MPRAGE and FLAIR image values for a specific location in position space (i.e. for each voxel). Distinct groupings by tissue type are readily identified.

**FIGURE 2:**
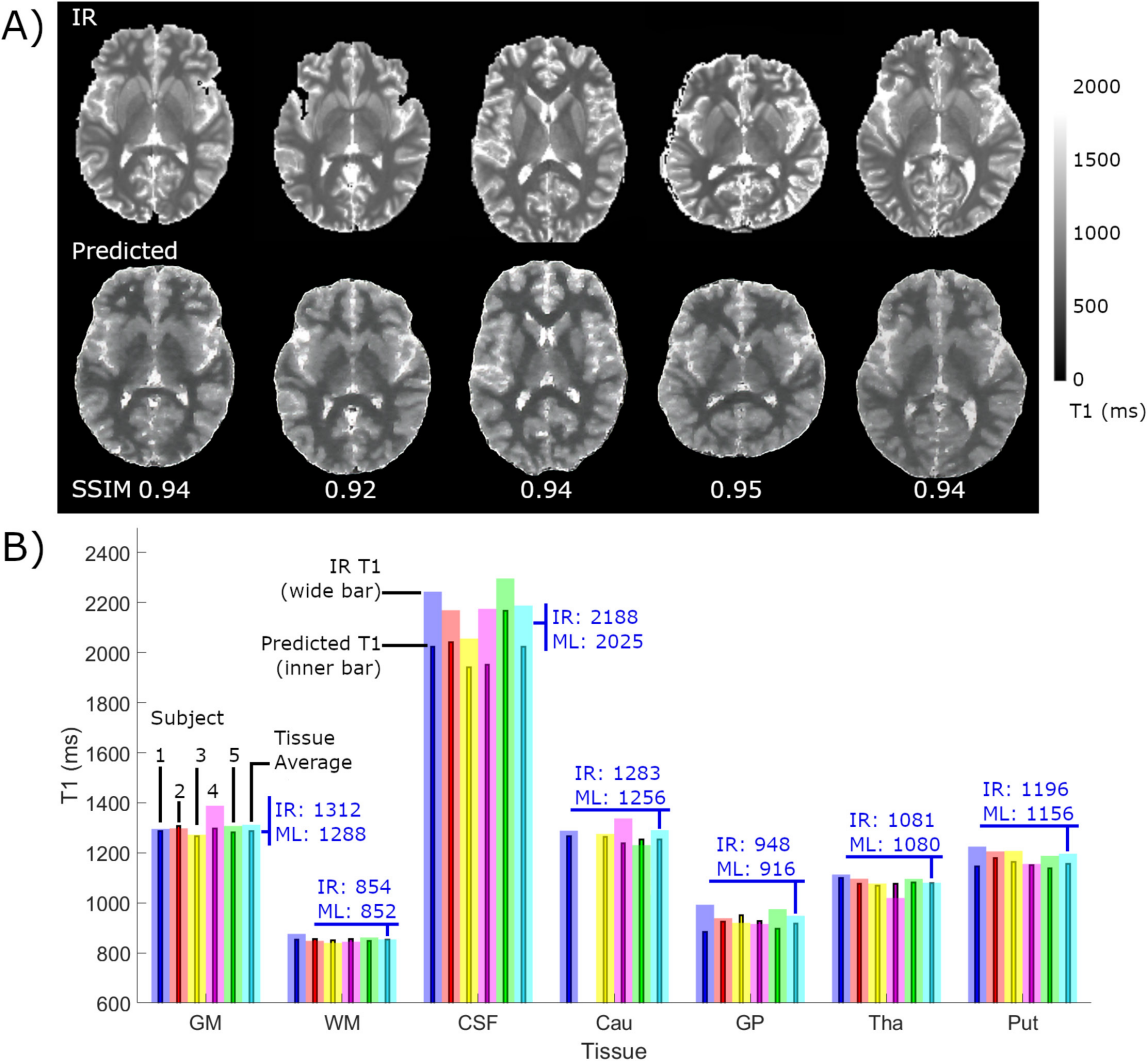
(A) Gold standard IR T1 maps (top row) compared to ML predicted T1 maps in five healthy subjects. The structural similarity index (SSIM) between IR and ML images are shown at bottom. (B) Mean tissue T1s for IR T1 maps (wide bars) versus ML T1 (narrow) for the same five healthy subjects in seven tissues. Subject averages are also shown as the last bar in each tissue set, with values shown in blue. Subject #2 did not present enough voxels for analysis in Cau due to slice location and is therefore not shown (empty bar).

IR FSE T1 maps (gold standard) and the predicted T1 maps from the ML are shown in Figure 2 for all five subjects, with difference analysis between IR and predicted shown in (B). Images have been denoised using a denoising convolutional neural network algorithm [28].

**FIGURE 3:**
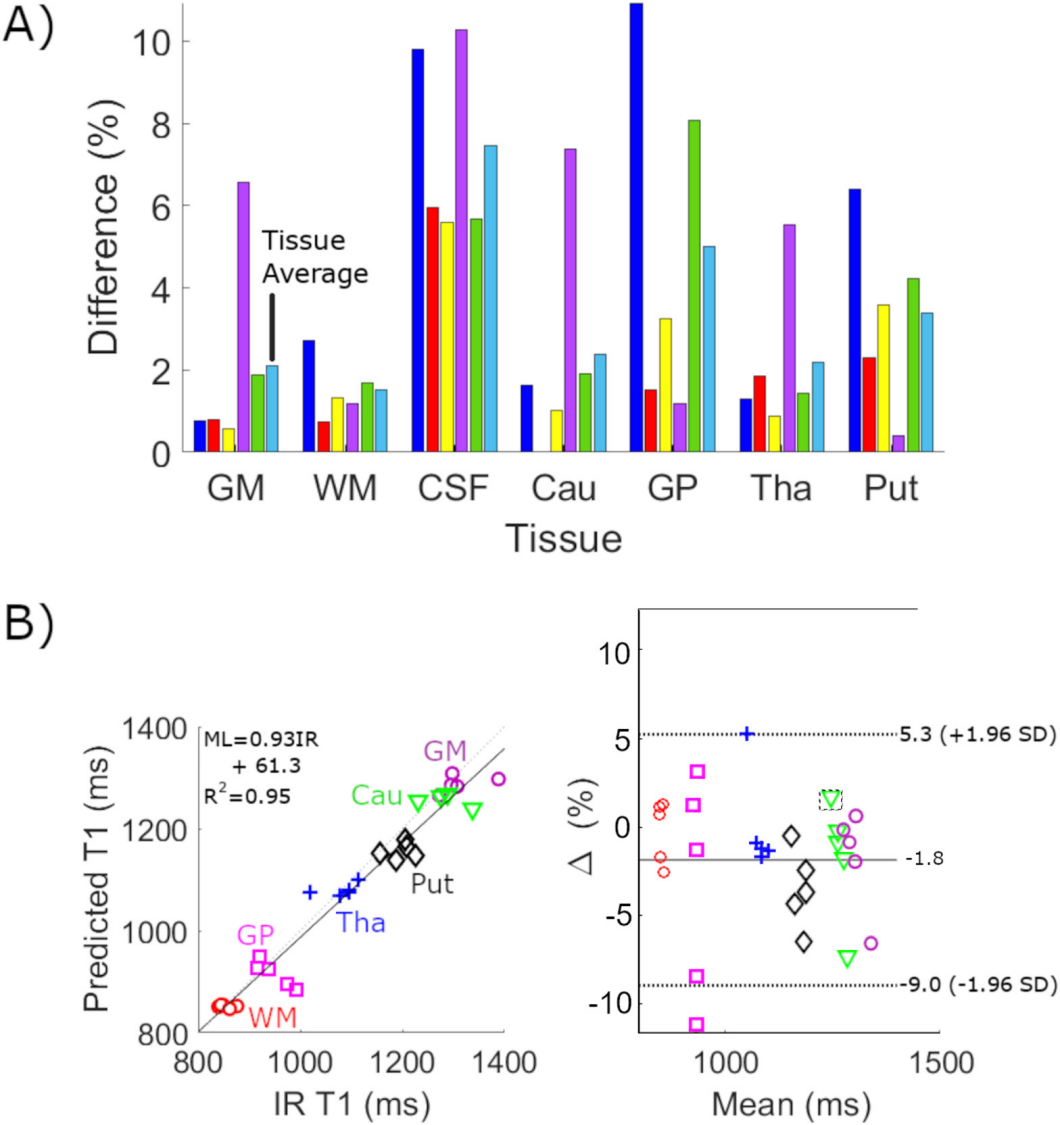
(A) Percent differences of T1 values between IR and ML methods for five healthy subjects. The average (of absolute voxel‐by‐voxel) percent difference for all subjects per tissue is shown as the final bar in each series. (B) Bland Altman analysis (excluding CSF) of the data in A for each tissue, with the correlation plot (left) and difference from the mean (BA plot, right). The equation to compute ML values from IR T1 values is shown at top left in the correlation plot based on the regression fit along with the *R* ^2^ metric. Confidence intervals are shown in the BA plot as dashed lines (+/−1.96 standard deviations), with the mean value as solid. The trend of the ML to underpredict T1 values is shown in the negative mean value and shift of the confidence intervals towards negative percentages.

Minor underprediction of T1 values occurs in all tissues, with the greatest discrepancy in CSF, likely due to registration and probability map errors between CSF and other tissues. SSIM values for all five subjects are greater than 0.92 indicating excellent comparison between IR and ML images. The underprediction trend is also visible in the ML T1 maps in Figure 2A as a general darkening of CSF. Further, the IR maps were interpolated to match MPRAGE resolution, and the thick slices used result in an averaging of axial spatial locations (blurring of T1 values). However, in general, excellent agreement between predicted and IR values is evident when comparing percent differences (absolute difference on a voxel‐by‐voxel basis), as shown in Figure 3.

In Figure 3A, average percent differences for all tissues were < 5%, with the greatest being 4.9 in GP (when excluding CSF). The remaining average differences were 2.1 (GM), 1.5 (WM), 2.4 (Cau), 2.2 (Tha) and 3.4 (Put). The correlation plot in Figure 3B (left) illustrates the underpredictive nature of the ML method compared to IR, with a regression slope 0.93, and a mean percentage difference of −1.8% (Figure 3B, right), with GP illustrating the largest variability.

### 3.2 Comparison of ML T1 Maps to MRF and MP2RAGE in MS Patients

**FIGURE 4:**
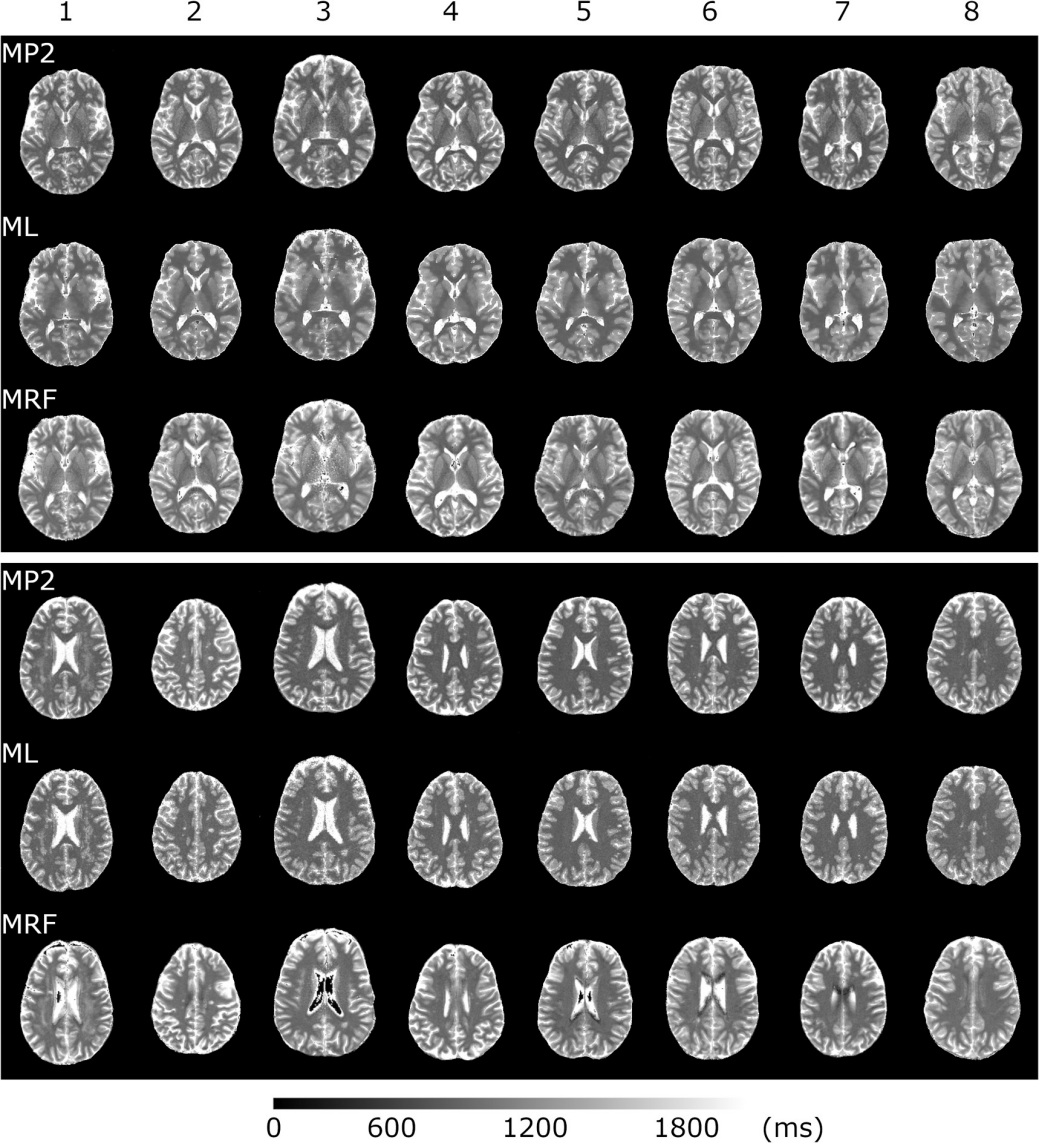
T1 maps for MP2RAGE (MP2; 1.0‐mm slice), the machine learning algorithm (ML; 1.0‐mm slice), and MRF (4.5‐mm slice) at deep GM (top three rows) and predominately WM (bottom) locations for all eight MS patients.

Predicted T1 maps and MP2RAGE and MRF maps are shown in Figure 4, with slice location of deep GM (top three rows) and predominantly WM in a superior brain location with typically greater lesion load (bottom).

**FIGURE 5:**
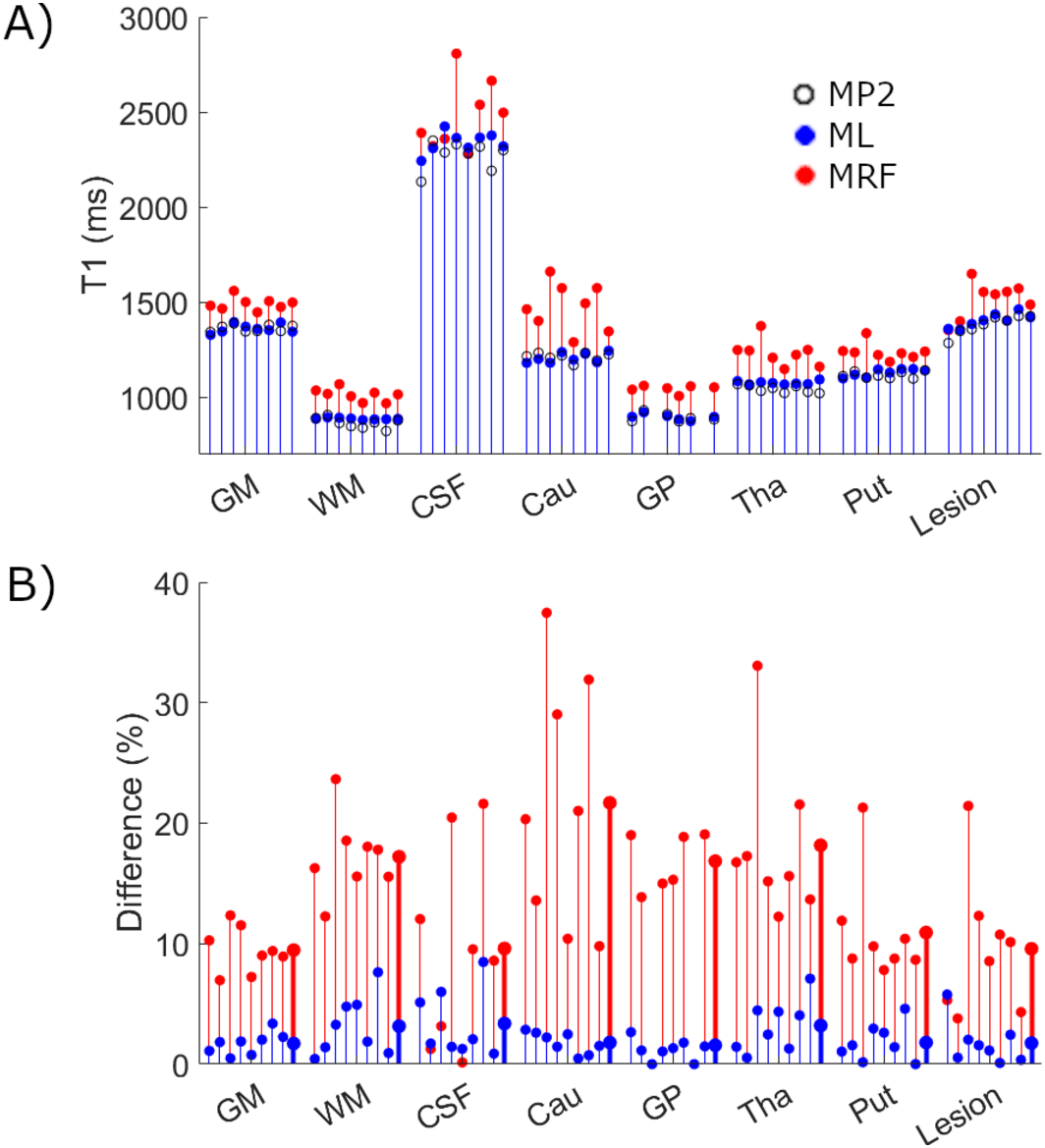
(A) Raw T1 values for the ML method (blue), MP2RAGE (black) and MRF (red). (B) Differences (absolute) between MP2RAGE and ML (blue) and MP2RAGE and MRF (red). Thick lines at the end of each tissue grouping represent the overall average absolute difference.

Visual inspection illustrates an equal shading of tissues in MP2RAGE and ML T1 maps, with a general brightening in all tissues in MRF (i.e. an increase in T1). This brightening leads to a loss of GM/WM contrast. Partial volume effects due to large voxels in MRF also tend to blur fine details, resulting in loss of smaller lesions as in the superior locations for patients 6 and 8. SSIM values comparing ML and MRF images ranged from 0.88 to 0.95 (average: 0.91), with slightly higher values occurring between ML and MP2RAGE (range: 0.89 to 0.95, average: 0.92). To further explore the T1 details, raw T1 values, T1 differences and Bland–Altman analysis are shown in Figure 5. Though no gold standard is available for the MS patient subset, two important comparisons can still be investigated. First, as the ML values were trained using MP2RAGE T1 as an output, the performance of ML should be similar to MP2RAGE with a similar ability to predict lesion T1s, with an overall underprediction of 1.8% across tissues as illustrated in the healthy volunteer validation study compared to gold standard IR FSE T1s. Secondly, the ability of MRF to predict T1 can be compared to ML T1s to investigate if MRF is able to improve on T1 values (i.e. overcome the 1.8% underprediction of ML and MP2RAGE). It should be noted that a direct comparison of T1 values per tissue with the healthy volunteer study cannot be achieved as lesions will artificially elevate T1s of GM and WM, depending on the tissue segmentation process. Most WM lesions were segmented as GM tissue, due to elevated FLAIR values not consistent with healthy WM tissue. Although this prevents the evaluation of normal appearing WM and GM (NAWM and NAGM, respectively) T1 as compared to healthy volunteers, the purpose of this study was to evaluate the ML method and not the actual T1s of tissues in MS. To compare lesion T1s directly, the additional lesion segmentation is shown in Figure 5.

Excellent agreement is apparent between MP2RAGE T1 values (black) and ML (blue) as shown in Figure 5B, with average absolute percent differences of 1.7 (GM), 3.2 (WM), 3.4 (CSF), 1.8 (Cau), 1.6 (GP), 3.2 (Tha) and 1.8 (Put). Additionally, lesion regions also show excellent agreement with an average percent difference of 1.7%. MRF did not perform as well compared to ML T1s with increased T1 values in each tissue, and an average percent difference across all tissues of 14% (minimum 9.5—GM, maximum 21.7—GP) as compared to ML T1s (Figure 5B). Bland–Altman analysis revealed similar trends, with WM displaying the greatest variability in T1s between MP2RAGE and ML, and values ranging from 7.4% (Patient 7—see Figure 5B) to −1.6% (Patient 2). GM had the least interpatient variability, which ranged from 2.3% to −1.3%.

## 4 Discussion

In this study, T1s produced from a kNN classification learner were compared to gold standard IR in healthy subjects and MP2RAGE and MRF in MS patients. The ML was trained using MPRAGE and FLAIR as inputs (standard clinical sequences) with MP2RAGE T1 values as outputs in an MS cohort consisting of 2.6 million total training points with varying tissue composition (including, lesions). The overall objective was the production of T1 maps using simple methods and readily available clinical data (with extension to retrospective studies lacking specific T1 mapping sequences). The comparison of ML output to gold standard IR FSE in healthy volunteers allowed validation of the ability of the ML to predict T1 values. Finally, evaluation of the ML in a second cohort of MS patients compared to MP2RAGE assessed the ability to predict T1s in diseased tissue, and whether the ML could perform similarly to MP2RAGE. Therefore, two main questions were investigated: (1) did the ML perform as well as MP2RAGE in predicted T1s? and (2) how accurate are ML T1s as compared to gold standard IR FSE?

In terms of T1 accuracy as assessed in healthy subjects, both IR FSE and ML‐produced T1 values within reported literature ranges [29, 30], with tissue values for ML T1s having a maximum deviation of < 5% from gold standard IR, and a global underprediction of T1 of −1.8%. GP had the highest variation in T1 values across the five healthy subjects. This variation may have been due to segmentation errors as GP can be difficult to delineate based on MPRAGE images, resulting in inconsistent partial voluming with neighbouring tissues across subjects.

Evaluation of the ML in the patient study included 6.9 million individual comparisons with MP2RAGE (eight MS patients) and resulted in excellent agreement (all average tissue deviations < 3.2%). The largest maximum deviation was 7.4% in a single patient (WM). As the ML and MP2RAGE have similar performance in MS patients, we can conclude that (1) the ML can be used to provide T1 values with similar accuracy to MP2RAGE, and (2) MP2RAGE will have similar T1 accuracy compared to IR FSE as the ML T1s. One of the strengths of the method is the compartmentalization of the data—each pixel is treated separately from all others in prediction. This allows the prediction to be performed on nondeformed datasets that may introduce errors when registered to specific atlas.

While MP2RAGE is specifically designed to compute T1s due to different IR times with a corresponding direct change in signal due to T1 relaxation between the two subset images, the ML was able to replicate similar T1s without a direct T1 dependence. In theory, a single MPRAGE image could be used to produce a T1 map by directly mapping pixel amplitudes to a known response dictionary. However, a normalization to match the dictionary would be required for every new scan. This would require measuring signal in two regions with large differences in T1 (e.g. GM and WM) and scaling the simulated MPRAGE response to fit these amplitudes. Due to pathology and geometrical differences between subjects, the normalization is difficult to automate and prone to error. In order to alleviate this dependence, typical qMRI methods use two or more acquired points (sets of images) to match decay curves and provide the necessary normalization [11, 31]. For this study, this second point requirement is fulfilled with T2‐weighted FLAIR, which is of general clinical importance and used extensively. However, the inclusion of FLAIR negates T2 independence of the T1 mapping method. As the FLAIR signal is proportional to both T1 and T2, this dependence is also incorporated in the ML and may have helped to delineate tissue type. Moreover, the complexity of qMRI fitting to Bloch‐simulated dictionaries is reduced by assuming specific parameters (such as T1 = 1 s for dual‐echo T2 mapping) or incorporating additional images (B_1_^+^, diffusion [32]) to account for known signal variations. In contrast, the learner inherently incorporates all fluctuations in the signal. Based on the data, it is not fully understood to what degree the ML can cope with incoherent variations (based on geometry such as B_1_^+^) as opposed to coherent signal change based on intensities. The segmentation process was used to provide more regional signal dependence and partly alleviates uncontrolled factors. While the ML may be agnostic to distinct signal dependence variations, it is expected that further refinement could be achieved by adding more information in the training stage. For example, regional differences based on B_1_^+^ variation should be investigated further to ensure ML predictability.

A statistical approach to T1 estimation was previously developed using conventional images and is based on similar data preparation as used in the kNN method [33]. This method also predicts T1s from individual voxels and showed an increase in accuracy when more imaging datasets were used. Consequently, the accuracy of T1 prediction using the machine learning approach in our study could be improved by using more images present in the protocol. Bloch equation modeling of T1 using clinical images has also been investigated [30] while simultaneously mapping T2, though not in the presence of pathology, and with mixed results of T1 values compared to gold standard IR. While Bloch equation modeling in T2 studies has produced excellent results [1, 13], it requires the complete simulation of all sequence parameters and timings, which we wanted to avoid to generalize the applicability of the ML method. However, a future direction could compare the Bloch simulation of MPRAGE and FLAIR to the ML T1 values.

The comparison to MRF is both informative and inconclusive. First, a more effective comparison requires further investigation in a larger cohort of patients and with a 3D MRF sequence [8, 34] with native 1‐mm^3^ voxels as opposed to the lower resolution 2D‐MRF approach that was available. Secondly, as MP2RAGE and ML T1s were comparable in the MS cohort, and ML compared well to the gold standard IR FSE, our implementation of 2D‐MRF with 4.5‐mm slices is therefore not as accurate as the ML and MP2RAGE in MS patients. In future assessments using any T1 quantification sequence, it is recommended to use an in‐house T1 standard (such as IR FSE) to ensure site‐specific repeatability due to the large range of literature T1 values [29].

Though 2.6 million input vectors and output intensities were stored for training, the data were only acquired from three MS subjects. In a pilot study, we examined ML differences due to training volume when changing the number of subjects from one to five (not shown). Significant improvements in the ML model were made when increasing subjects to three as compared to a single subject. Beyond three subjects, additional training data did not improve the results, though further data inclusion could potentially help improve difficult areas like the GP. Including further data also increases the time required for training and prediction, with additional constraints on hardware. A larger amount of subjects could be incorporated into the training data by keeping the total number of training points constant (e.g. 2.6 million) while randomly selecting points from > 3 subjects. Further, after training is accomplished, changes to sequence parameters (MPRAGE and FLAIR) used as input image data to predict T1 maps may produce undesired results. As the learner is based on voxel intensity, any sequence change after training may produce altered voxel intensities in the image sets, thereby lessening the accuracy of the prediction and requiring retraining. Additionally, specific diseases altering the GM/WM intensities differently than MS would require retraining.

In conclusion, this study presents a simple T1 mapping method based on standard clinical images only and simple machine learning techniques that are easily approachable, with T1 images agreeing well with gold standard IR T1 maps. This allows T1 map creation from retroactive and current studies that do not use specialized T1 mapping sequences. The ML‐produced T1s have excellent agreement with MP2RAGE in MS patients, with similar values across multiple tissues including lesions. Consequently, the ML can be used as a similar substitute for T1 map creation when dedicated T1 mapping sequences are not available.

## Conflicts of Interest

The authors declare no conflicts of interest.

## Data Availability

Research data not shared.
